# Point-of-care AI-enhanced novice echocardiography for screening heart failure (PANES-HF)

**DOI:** 10.1038/s41598-024-62467-4

**Published:** 2024-06-12

**Authors:** Weiting Huang, Tracy Koh, Jasper Tromp, Chanchal Chandramouli, See Hooi Ewe, Choon Ta Ng, Audry Shan Yin Lee, Louis Loon Yee Teo, Yoran Hummel, Feiqiong Huang, Carolyn Su Ping Lam

**Affiliations:** 1 National Heart Centre Singapore, 5 Hospital Drive, Singapore, 169609 Singapore; 2 Duke-NUS Medical School, Singapore, Singapore; 3 Saw Swee Hock School of Public Health, National University of Singapore, and National University Health System Singapore, Singapore, Singapore; 4 Us2.ai, Singapore, Singapore

**Keywords:** Artificial intelligence, Heart failure screening, Novice ultrasound, Heart failure diagnostic pathway, Computational biology and bioinformatics, Cardiology

## Abstract

The increasing prevalence of heart failure (HF) in ageing populations drives demand for echocardiography (echo). There is a worldwide shortage of trained sonographers and long waiting times for expert echo. We hypothesised that artificial intelligence (AI)-enhanced point-of-care echo can enable HF screening by novices. The primary endpoint was the accuracy of AI-enhanced novice pathway in detecting reduced LV ejection fraction (LVEF) < 50%. Symptomatic patients with suspected HF (N = 100, mean age 61 ± 15 years, 56% men) were prospectively recruited. Novices with no prior echo experience underwent 2-weeks’ training to acquire echo images with AI guidance using the EchoNous Kosmos handheld echo, with AI-automated reporting by Us2.ai (AI-enhanced novice pathway). All patients also had standard echo by trained sonographers interpreted by cardiologists (reference standard). LVEF < 50% by reference standard was present in 27 patients. AI-enhanced novice pathway yielded interpretable results in 96 patients and took a mean of 12 min 51 s per study. The area under the curve (AUC) of the AI novice pathway was 0.880 (95% CI 0.802, 0.958). The sensitivity, specificity, positive predictive and negative predictive values of the AI-enhanced novice pathway in detecting LVEF < 50% were 84.6%, 91.4%, 78.5% and 94.1% respectively. The median absolute deviation of the AI-novice pathway LVEF from the reference standard LVEF was 6.03%. AI-enhanced novice pathway holds potential to task shift echo beyond tertiary centres and improve the HF diagnostic workflow.

## Introduction

Due to rapidly ageing societies, the prevalence of heart failure (HF) is increasing worldwide^1^. Advances in HF therapeutics have significantly improved patient outcomes^2^. Delayed initiation of guideline-recommended therapies for HF can lead to unnecessary hospitalisations and deaths^3,4^. Therefore, early diagnosis and subsequent treatment are critical^5–10^.

Echocardiography, the primary method for evaluating the HF subtype, guiding HF treatment decisions and counselling, requires trained sonographers and cardiologists to acquire and interpret images^11^. Unfortunately, the lack of highly trained sonographers and cardiologists is a rate-limiting step in the HF diagnostic care pathway. This delays diagnosis and treatment access, deprives patients of the benefits of proven therapies and leads to worse patient outcomes^12–15^.

Established guidelines have demonstrated a difference in treatment strategies between HF with reduced ejection fraction (< 40%) and HF with preserved ejection fraction (≥ 50%)^16^, while studies and post hoc analyses suggested that patients with HF with mildly reduced ejection fraction (40–49%) benefit from therapies similar to their counterparts with reduced ejection fraction^17–20^. Hence, for purposes of initiation of treatment, knowledge of the presence of left ventricular systolic dysfunction, defined as left ventricular ejection fraction (LVEF < 50%) is vital. Moreover, the first HF presentation for most patients is usually to a primary care clinic, where without appropriate evaluation tools, diagnosis may be missed and treatment delayed^14,21^. With the increased uptake of point-of-care (POC) ultrasound devices in primary care clinics^22,23^, there is opportunity to improve HF care using artificial intelligence (AI).

Previous studies demonstrated that untrained novices could acquire echocardiographic images of sufficient quality after only limited training^24,25^. However, image interpretation was the rate-limiting step, requiring trained cardiologists or sonographers. Advances in deep learning have shown the potential of automated algorithms to interpret cardiac structural and functional information effectively^26–28^. Combined with POC echocardiographic devices, these algorithms could empower novices to perform echocardiographic examinations, thus overcoming barriers to access, allowing prompt initiation of treatment and reducing delays in the HF diagnostic pathway^29,30^.

Therefore, this study investigated the diagnostic accuracy of an end-to-end package by novice and AI for detecting left ventricular systolic dysfunction, without intervention from trained sonographers or cardiologists.

## Methods

### Study design, study population and sample size

In this cross-sectional study, all patients aged ≥ 21 years with a at least one HF symptom (pedal oedema, New York Heart Association (NYHA) II-III effort limitation, orthopnoea, breathlessness), were eligible for inclusion. Patients were recruited over a 6-month period.

Eligible patients presenting to the cardiac imaging laboratory at the National Heart Centre Singapore for physician-ordered clinical echocardiogram for investigation or follow up for HF were approached for recruitment. Recruited patients underwent standard cart-based sonographer-performed, cardiologist-reported echocardiogram as per clinical practice, and novice-performed AI-supported POC echocardiogram.

Pregnant women were excluded. All patients provided written informed consent before participation. Ethics approvals were obtained from the local institutional review committee (Singhealth Centralised Institutional Review Board) for this study, and this study conformed to the ethical guidelines in the Declaration of Helsinki.

The sample size was calculated based on previously published standard deviations^31^ and the assumption that the study population’s mean left ventricular ejection fraction (LVEF) was 50%, with an equivalence region of 5%^32^. The sample size required for non-inferiority with 90% statistical power was at least 79 patients. We considered potential difficulties in image acquisition, hence 100 patients were recruited for this study.

### Novice selection and training

A clinical study coordinator (she/her) without prior sonography experience was invited to participate. She was trained to identify cardiac structures and key landmarks on ultrasound using the 2015 American Society of Echocardiography Chamber Quantification^33^. She was then given the POC ultrasound device (EchoNous Kosmos) and was attached to the cardiac imaging laboratory under the supervision of sonographers for 2 weeks. In the first week, she observed the sonographers in image acquisition. She was allowed hands-on practice with guidance in acquiring seven standard views (apical 4,3 and 2 chambers and parasternal long, short axis at the mitral, mid and apical levels) for LVEF assessment. In the second week, after independently acquiring the seven standard images using the POC ultrasound device in 10 patients, she started recruitment.

### POC device and software

This study used the Torso-One cardiac probe and the Kosmos Bridge tablet with image processing capabilities. The AI TRIO, the background AI image acquisition program, provides real-time image guidance on fanning, rotating, and repositioning the probe to achieve better image quality. It contains anatomic labelling of the structures in the image and grades the image in real time, so users know via a green light indicator when they have acquired an adequate picture.

Us2.ai, an AI-driven vendor-independent cardiac ultrasound image processing platform, was used for image interpretation. This software’s external and United States Food and Drug Administration (FDA) validation results were published previously^31^. Briefly, Us2.ai is a deep learning-based workflow which automates the entire process by rapidly analysing the digital imaging and communications in medicine (DICOM) files of a patient’s echocardiographic exam without the need for human intervention, from the classification of views, identifying cardiac phases to giving readouts of relevant cardiac measurements such as the LVEF.

During the study, the novice attempted to capture seven standard views (apical 4,3 and 2 chambers and parasternal long, short axis at the mitral, mid and apical levels) for LVEF interpretation. The LVEF readout by the AI followed the priority dependent on image quality, biplane followed by monoplane.

### Study outcomes

The primary outcome of this study was the accuracy of AI-enhanced novice-performed POC echocardiogram and AI-interpreted LVEF (AI-enhanced novice pathway) to detect a reduced LVEF < 50%. The cardiologist-reported LVEF based on the sonographer-acquired traditional cart-based clinical echocardiogram was the reference standard.

Secondary outcomes were the yield and the learning curve of a novice performing POC cardiac ultrasound. The yield was defined as the proportion of POC ultrasound exams with an AI-measurable LVEF on any of the views using the number of cart-based cardiologist reported LVEFs as the denominator. The learning curve was assessed by the time taken for image capture for each study over the study duration. The time taken for image capture was defined as the difference between the first and last image recorded for each study.

### Statistical analysis

Continuous variables with a normal distribution were presented as means ± one standard deviation. Variables with a non-normal distribution were expressed as median and interquartile range. Binary variables were expressed as a number count and proportion. The accuracy by area under the curve (AUC), sensitivity, specificity, positive predictive and negative predictive values were tabulated. The agreement between AI-enhanced novice pathway and reference standard LVEF was assessed by percent agreement and Cohen’s Kappa. We also calculated the mean absolute error (MAE), root mean square error, median absolute and relative (percentage) deviation, and the Pearson correlation coefficient, *r*, for AI- enhanced novice pathway versus reference standard measurements.

The learning curve of the novice was calculated by tabulating the rolling cumulative average of scan durations by chronological patient order and fitting a Lowess curve of time taken to complete a scan against the chronological patient number recruited across time. The learning rates were tabulated for the first 20, next 20 to 40, and 40 to 60 patients, using Wright’s model^34^ where Y = aX^b^; Y = the cumulative average time per study, X = the cumulative number of studies performed, a = time required for the first study and b = slope of the function when plotted on log–log paper, which is also the log of the learning rate/log of 2.

All analyses were performed using Stata statistical software (version 14.0, Stata Corporation, College Station, Texa ds, USA). All statistical analyses were conducted at the significance level of 0.05 and all tests were two-tailed whenever appropriate.

### Ethical approval

Ethics approvals were obtained from the local institutional review committee (Singhealth Central Institutional Review Board) for this study, and this study conformed to the ethical guidelines in the Declaration of Helsinki.

## Results

### Demographics

Out of 100 participants, 56 (56%) were men, and the average age of the cohort was 61.2 ± 15.0 years. Forty (40%) patients had a history of HF, while others had symptoms suggestive of HF, including peripheral edema (54%), orthopnea (9%), and breathlessness (48%). Ninety-five patients were in sinus rhythm, four were in atrial fibrillation, and one had a paced rhythm (Table 1). Ninety-six studies in the AI-enhanced novice pathway had sufficient image quality for AI interpretation to produce an LVEF readout; hence the yield was 96%.

**Table 1:** Baseline characteristics.

Demographics	(n = 100)
Age (years)	61.2 ± 15.0
Male	56 (56%)
Race	
Chinese	75 (75%)
Malay	8 (8%)
Indian	13 (13%)
Others	4 (4%)
Hypertension	55 (55%)
Diabetes mellitus	27 (27%)
Dyslipidemia	64 (64%)
Systolic blood pressure (mmHg)	130 ± 19
Diastolic blood pressure (mmHg)	75 ± 8
Current smoker	13 (13%)
History of heart failure	40 (40%)
History of coronary artery disease/ischemic heart disease	29 (29%)
Symptom of leg swelling	23 (23%)
Symptoms of orthopnea	12 (12%)
Symptoms of breathlessness	
None	39 (39%)
On exertion	48 (48%)
At rest	1 (1%)
Anytime	12 (12%)
New York Heart Association Class	
I	39 (39%)
II	50 (50%)
III	6 (6%)
IV	5 (5%)
Heart rhythm	
Sinus	94 (94%)
Atrial fibrillation	4 (4%)
Atrial Ectopics	1 (1%)
Paced rhythm	1 (1%)
Mean LVEF (%)	54.7 ± 11.7
LVEF < 50%	27 (27%)

*LVEF* left ventricular ejection fraction.

### Performance of AI-enhanced novice pathway in predicting LVEF < 50%

The sensitivity, specificity, positive predictive and negative predictive values of the AI-enhanced novice pathway in detecting LVEF < 50% were 84.6%, 91.4%, 78.5% and 94.1% respectively. The AUC of the AI-enhanced novice pathway in detecting LVEF < 50% was 0.880 (95% CI 0.802, 0.958). The agreement between the reference standard and AI-enhanced novice pathway in the detection of LVEF < 50% was percent agreement 0.895, Cohen Kappa 0.742 ± 0.101.

**Figure 1:**
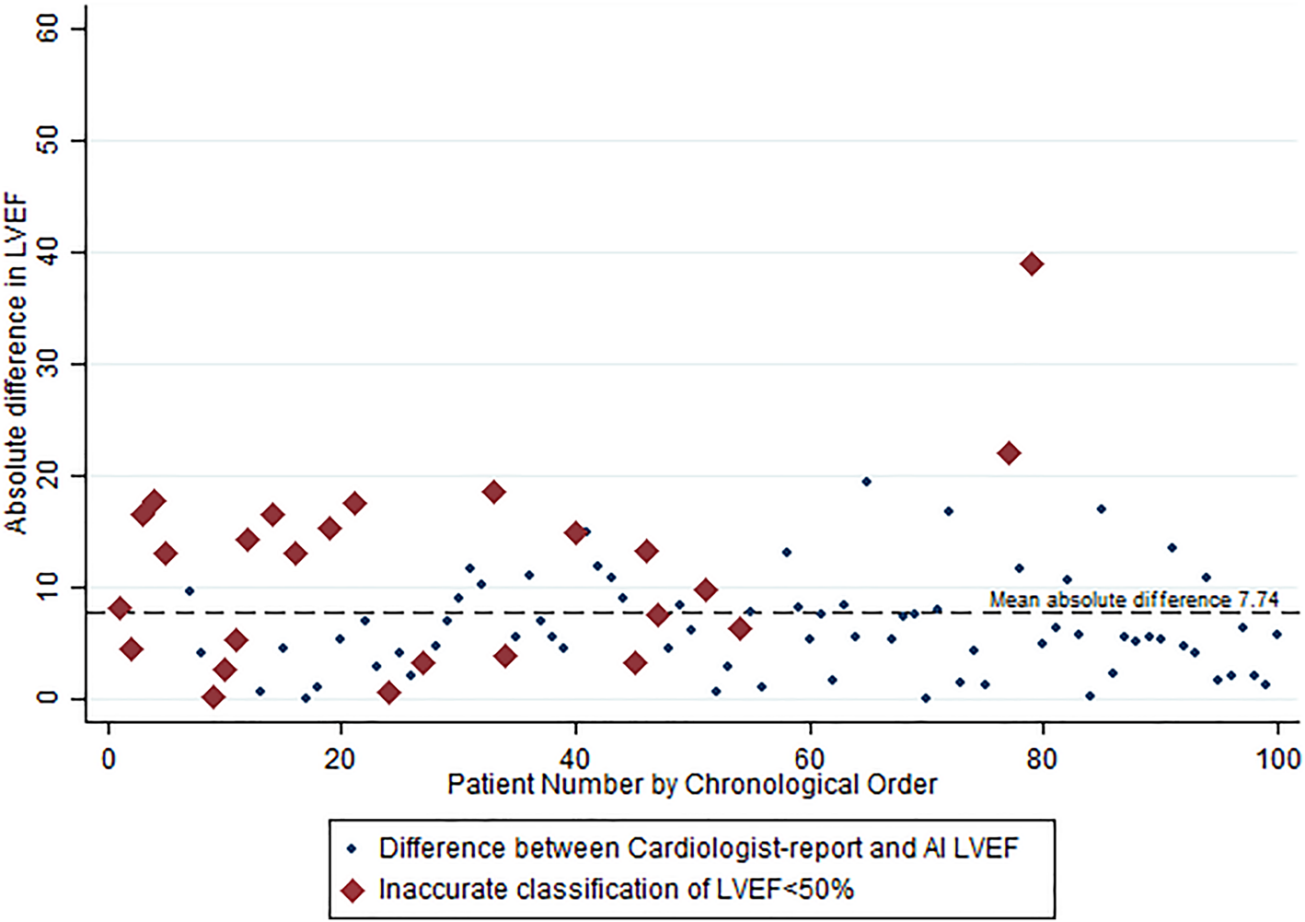
Absolute difference between reference standard and AI-enhanced novice pathway LVEF.

The correlation between the reference standard LVEF and the AI-novice LVEF was 0.713. When comparing actual LVEF values by the AI-enhanced novice pathway and reference standard, the median absolute deviation was 6.03%, with a median relative percentage deviation of 10.9%. The root mean square error of the AI-enhanced novice pathway against the reference standard was 8.31, with a mean absolute error (MAE) of 7.74. The absolute difference in LVEF between reference standard and AI-enhanced novice pathway, and subjects incorrectly classified at the individual patient level is shown in Fig. 1.

#### Learning curve of novice

**Figure 2:**
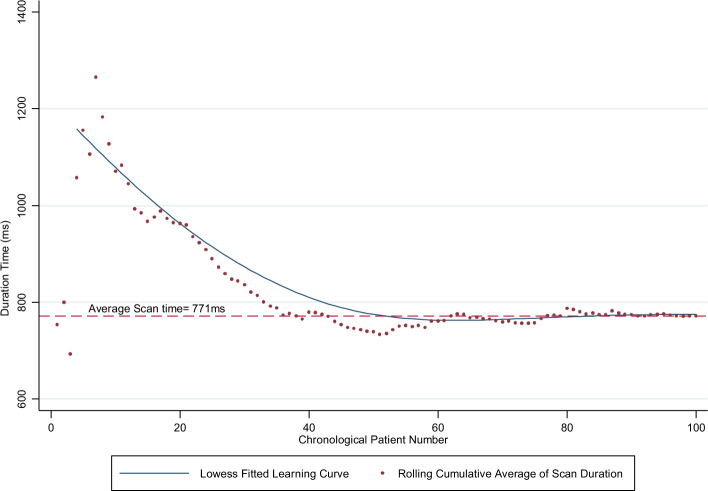
Learning curve by cumulative average of scan durations.

The mean time required for the layperson to perform an AI-guided handheld echocardiogram was 12 min and 51 s. The learning rate for the first 20 patients was 60.2%, 30.4% for patients 20 to 40, and plateaued at 3.5% for patients 40 to 60 (Fig. 2).

There was no significant difference in accuracy by AUC in predicting LVEF < 50% between the first 40 and next 60 patients (p = 0.652). There was also no difference in the LVEF mean absolute deviation between both pathways in the first 40 and next 60 patients (p = 0.468).

## Discussion

This study demonstrated that an AI-enhanced novice with no prior sonography background could successfully perform LVEF assessments for patients with suspected or current HF. The novice obtained sufficient AI-interpretable images in 96% of studies performed with a high AUC of more than 0.80. This is important as we view HF across a spectrum of ejection fraction, with individualised therapies for each ejection fraction category^35^. Apart from diagnosing HF, the ejection fraction is an important marker for therapy initiation. Our study timely demonstrates that a new workflow can significantly increase accessibility to LVEF measurements.

The AI-enhanced novice pathway offers a more sensitive and objective alternative for physicians to confidently initiate appropriate guideline directed medical therapy based on LVEF, especially in primary care clinics, before confirmatory echocardiograms are performed. In contrast NT-proBNP, a common initial diagnostic test for patients presenting with HF symptoms, is confounded by common comorbidities such as atrial fibrillation, renal dysfunction and obesity^36^, and is unable to give the LVEF readout for therapy initiation. Given its high negative predictive value, the AI-enhanced novice pathway is also a valuable screening tool for at-risk Stage B heart failure patients with comorbid conditions, but do not exhibit symptoms of heart failure yet.

Prior studies, which largely compared differences in measurements between manual readers and automated AI based on the same sonographer-acquired images, reported an MAE range in LVEF to be 6–10%^37^ and median percentage deviation of automated AI LVEF was approximately 6%^26^. Although the novice acquired images in this study, the MAE was favourably comparable at 6.03% with a median percentage deviation of 10.9%.

We also looked at the performance of the novice in the real-world situation where patients of different habitus and acoustic windows were recruited, instead of a small controlled patient cohort in the previous study by Schneider et al.^25^. We achieved comparable LVEF agreement of 0.713 between AI and human expert (versus correlation coefficient r 0.79–0.92) and a similar incidence (96% versus 91%) of diagnostic quality images. Further information on the learning curve of the novice also provides background to determine adequacy of training before novices reach their optimal scan time for image acquisition.

Additionally with the help of AI, a relatively short time was taken to perform the scans (approximately 12 min); hence novices using POC ultrasound devices to acquire images for LVEF assessment in suspected HF patients is a feasible, implementable service in busy primary care settings. Use of AI in echocardiogram reduces scanning time and allows almost instantaneous reporting of results. The LVEF is both diagnostic and prognostic in assessing a wide variety of cardiac conditions, such as coronary artery disease, arrhythmias, syncope, suspected HF etc^38^. This AI-enhanced novice pathway holds potential for new workflows to improve resource allocation and increase diagnostic throughput without sacrificing accuracy. This is invaluable in addressing real-world shortage of echocardiogram services^39^ by increasing accessibility to LVEF assessments in conditions where only LVEF is required for screening and therapy initiation (e.g. non-valvular atrial fibrillation, palpitations, monitoring during chemotherapy) and in turn improving wait times for conditions where detailed sonographer echocardiogram assessments are required (e.g. valvular heart disease, infective endocarditis). Integrating both the AI-enhanced novice pathway and traditional sonographer-cart-based workflows will enable cardiac imaging laboratories to meet the increasing demands for cardiac imaging better and allow physicians to provide better, timely care for cardiac patients.

### Limitations

This study has multiple limitations. First, our sample of a small Asian population could limit generalisation as real-world patients with HF. Next, our data consists of patients with sinus rhythm (94%) – it would be helpful to assess the system under stress with arrhythmias. Finally, it is unclear if suboptimal image quality in 4% of our population is attributable to end-user or AI, as only one AI-novice pair was included in this study. Additionally, as a significant proportion of reference LVEF was reported by visual estimation, we did not manage to compare the LV volumes reported by both pathways.

## Conclusion

In conclusion, our study affirms AI's role in empowering a novice to perform POC ultrasound to obtain accurate diagnostic information to diagnose HF. This augments the supply of echocardiogram services by minimising the sonographic expertise required in the traditional cart-based setting. Future studies involving a larger population and integrating this AI-enhanced novice pathway are underway.

## Data Availability

The datasets generated during and/or analysed during the current study are available from the corresponding author on reasonable request.

## Abbreviations

AUC: Area under the curve
DICOM: Digital imaging and communications in medicine
FDA: United States food and drug administration
HF: Heat failure
LVEF: Left ventricular ejection fraction
MAE: Mean absolute error
NYHA: New York Heart Association
NT-proBNP: N-terminal pro–B-type natriuretic peptide
POC: Point-of-care
